# Computing Power and Sample Size for the False Discovery Rate in Multiple Applications

**DOI:** 10.3390/genes15030344

**Published:** 2024-03-07

**Authors:** Yonghui Ni, Anna Eames Seffernick, Arzu Onar-Thomas, Stanley B. Pounds

**Affiliations:** Department of Biostatistics, St. Jude Children’s Research Hospital, Memphis, TN 38105, USA; yonghui.ni@stjude.org (Y.N.); anna.seffernick@stjude.org (A.E.S.); arzu.onar@stjude.org (A.O.-T.)

**Keywords:** false discovery rate, power, sample size, multiple testing, proportion of true null hypotheses

## Abstract

The false discovery rate (FDR) is a widely used metric of statistical significance for genomic data analyses that involve multiple hypothesis testing. Power and sample size considerations are important in planning studies that perform these types of genomic data analyses. Here, we propose a three-rectangle approximation of a *p*-value histogram to derive a formula to compute the statistical power and sample size for analyses that involve the FDR. We also introduce the R package *FDRsamplesize2*, which incorporates these and other power calculation formulas to compute power for a broad variety of studies not covered by other FDR power calculation software. A few illustrative examples are provided. The *FDRsamplesize2* package is available on CRAN.

## 1. Introduction

The false discovery rate (FDR) is now a widely used metric of statistical significance for exploratory data analyses that perform a very large number of hypothesis tests. Benjamini and Hochberg introduced the FDR and proposed a *p*-value adjustment procedure to control it under certain conditions [1]. Subsequently, many procedures to estimate or control the FDR and similar metrics of statistical significance have been developed [2]. Most of these later procedures improve power by incorporating an estimate ${\hat{\pi }}_{0}$ of the proportion $\pi_{0}$ of hypothesis tests that have a true null hypothesis. Others compute ${\hat{\pi }}_{0}$ as the height of the rightmost bin of a histogram of all *p*-values [3,4,5]; Pounds and Cheng compute ${\hat{\pi }}_{0}$ as the minimum of 1 or twice the mean of all *p*-values [6]; others have elaborate methods to compute ${\hat{\pi }}_{0}$. These FDR methods have found widespread application in the analysis of genomic data in exploratory analyses that test the association of each of many genomic features with a phenotype or other biological condition of interest.

The widespread use of these methods has fueled the development of several methods to compute power and sample size for studies that use the FDR to determine statistical significance. Most of these methods compute power and sample size for two-group comparisons. Jung proposed a particularly elegant method based on a simple equation involving the desired FDR τ, the desired average power 1−β of tests with a false null [7], the proportion $\pi_{0}$ of tests with a true null, and the unadjusted *p*-value threshold α used to declare statistical significance [8]. Jung solves this equation for α and then finds the sample size n to provide 1−β average power to the tests with a false null hypothesis and provides specific details for using a t-test to compare the means of many variables across two groups [7].

Pounds and Cheng expand on Jung’s [7] approach by considering that, in practice, the estimate ${\hat{\pi }}_{0}$ is used instead of $\pi_{0}$ itself to determine statistical significance [9]. The estimate ${\hat{\pi }}_{0}$ is a function of the data and thus is dependent on sample size. Pounds and Cheng derive a formula for this relationship, incorporate it into Jung’s equation [7], and then develop an iterative procedure that incorporates this consideration into sample size calculations [9]. They also provide software to perform these calculations, using one-way ANOVA to compare the means of many variables across three or more groups. This iterative procedure can be computationally expensive. In some cases, the iterative procedure yields the same sample size calculation as Jung’s equation because ${\hat{\pi }}_{0}$ converges to $\pi_{0}$ as the sample size increases.

Here, we propose a *p*-value histogram framework to develop a simplified approach to consider the estimation of $\pi_{0}$ in sample size calculations. We incorporate this approach into the *FDRsamplesize2* R software package (version: 0.2.0) for power and sample size calculations. The *FDRsamplesize2* package computes power and sample size for several types of scientific questions that are not covered by other packages. The *FDRsamplesize2* package can perform these calculations in settings where all hypotheses are evaluated with one of the following tests: two-proportions z-test, a two-group comparison of Poisson data, comparison of negative binomial data, one-sample or two-sample *t*-test, *t*-test for non-zero correlation, Fisher’s exact test, signed rank test, sign test, rank sum test, ANOVA, and single-predictor Cox regression. Our software also computes power and sample size using Jung’s formula [7] and our *p*-value histogram formulas. It also computes power for using the Benjamini and Hochberg *p*-value adjustment method [1] (which does not estimate ${\hat{\pi }}_{0}$ for FDR control).

## 2. Background

Consider a setting that involves performing a very large number of hypothesis tests. A proportion $\pi_{0}$ of these tests have a true null hypothesis, and thus, the remaining proportion $1-\pi_{0}$ of tests have a false null hypothesis. We want to design a study so that we have adequate statistical power to declare significance for 1−β of the $1-\pi_{0}$ of tests with a false null hypothesis (average power of 1−β across tests with a false null hypothesis) while controlling the FDR at a fixed, pre-specified level τ.

For this problem, Jung noted that:

(1) $$\tau =\frac{\pi_{0}\alpha }{\pi_{0}\alpha +\left(1-\pi_{0}\right)\left(1-\beta \right)}$$

accurately approximates the relationship among the FDR τ, the proportion $\pi_{0}$ of tests with a true null hypothesis, the (unadjusted) *p*-value threshold α used to declare statistical significance, and the average power 1−β of all tests with a false null hypothesis at the α level [7]. Solving for α yields a fixed *p*-value threshold:

(2) $$\alpha =\frac{\tau \left(1-\pi_{0}\right)\left(1-\beta \right)}{\pi_{0}\left(1-\tau \right)}$$

that achieves the desired average power 1−β and desired FDR control τ, given the proportion $\pi_{0}$ of tests with a true null hypothesis. One can then use the power calculation formula for the statistical method of interest to compute the sample size n necessary for tests with a false null hypothesis to have an average power $\left(1-\beta \right)$ at the α level.

Pounds and Cheng noted that in practice:

(3) $$\hat{\tau }=\frac{{\hat{\pi }}_{0}\alpha }{\pi_{0}\alpha +\left(1-\pi_{0}\right)\left(1-\beta \right)}$$

is used to determine statistical significance, where all observed *p*-values are used to compute an estimate ${\hat{\pi }}_{0}$ of $\pi_{0}$ as well as local FDR estimates $\hat{\tau }$ as a function of the *p*-value threshold α [9]. The estimate ${\hat{\pi }}_{0}$ appears only in the numerator of this equation because the FDR adjustment procedures only use ${\hat{\pi }}_{0}$ in the numerator. In FDR adjustment procedures, the empirical distribution function (EDF) of all *p*-values is used to estimate the denominator; thus, ${\hat{\pi }}_{0}$ is not needed in the denominator. In sample size calculations, the denominator is the average cumulative distribution function (CDF) of all *p*-values, which is a function of the conjectured value $\pi_{0}$. The denominator is the average CDF of all tests, mathematically represented with separate terms for the $\pi_{0}$ of tests with a true null and the $1-\pi_{0}$ tests with a false null to illuminate the relationship between FDR and average power 1−β. Recall that the statistical power of a test at the α level is the CDF of the *p*-value for that test. Therefore, ${\hat{\pi }}_{0}$ and $\hat{\tau }$ themselves also depend on the average power 1−β of the tests with a false null hypothesis, and thus, the sample size. Pounds and Cheng derive the formula for the expected value $E\left({\hat{\pi }}_{0}|n,\theta \right)$ of ${\hat{\pi }}_{0}$ as a function of the sample size n and parameter vector θ (effect sizes of all tests), and then develop an iterative procedure that incorporates this consideration into the sample size calculations [9]. This iterative procedure adjusts for the fact that data-based estimates are used in practice. In some cases, this procedure obtains larger sample sizes than Jung’s formula because $E\left({\hat{\pi }}_{0}|n,\theta \right)\geq \pi_{0}$ for most methods to compute ${\hat{\pi }}_{0}$. In other cases, this procedure obtains the same sample size as Jung’s formula because $E\left({\hat{\pi }}_{0}|n,\theta \right)\to \pi_{0}$ as n→∞. Nevertheless, the iterative procedure is computationally burdensome. Thus, a more straightforward and less computationally complex approximation can be useful.

Following [7], let us consider the approximate relationship:

(4) $$\tilde{\tau }=\frac{{\tilde{\pi }}_{0}\alpha }{\pi_{0}\alpha +\left(1-\pi_{0}\right)\left(1-\beta \right)\;\;\;\;\;}$$

where ${\tilde{\pi }}_{0}$ is used to compute $\tilde{\tau }$. For example, ${\tilde{\pi }}_{0}=\pi_{0}$ in Jung’s equation above. Also, the Benjamini and Hochberg method operationally uses ${\tilde{\pi }}_{0}=1$ to obtain $\tilde{\tau }$ [1]. Using ${\tilde{\pi }}_{0}=1$ in the equation above and solving for α yields:

(5) $$\alpha_{BH}=\frac{\tilde{\tau }\left(1-\pi_{0}\right)\left(1-\beta \right)}{1-\tilde{\tau }\pi_{0}}$$

as the *p*-value threshold determined by using the Benjamini and Hochberg procedure to control the FDR at τ in a setting with 1−β average power of tests with a false null hypothesis [1]. One can then use the power formula for the tests with false null hypotheses to determine the sample size needed to achieve an average power of 1−β at the $\alpha_{BH}$ level.

Histograms of *p*-values are a useful component of several FDR control procedures. In the q-value procedure, Storey estimates $\pi_{0}$ by the height of a histogram bar for *p*-values between 0.95 and 1.00 [3]. Nettleton et al. and Pounds et al. also use histograms to compute estimates of $\pi_{0}$ [4,5]. This suggests that a histogram approximation to the *p*-value distribution may help in developing a useful framework for computing power and sample size for controlling the FDR in applications involving many hypothesis tests.

**Figure 1:**
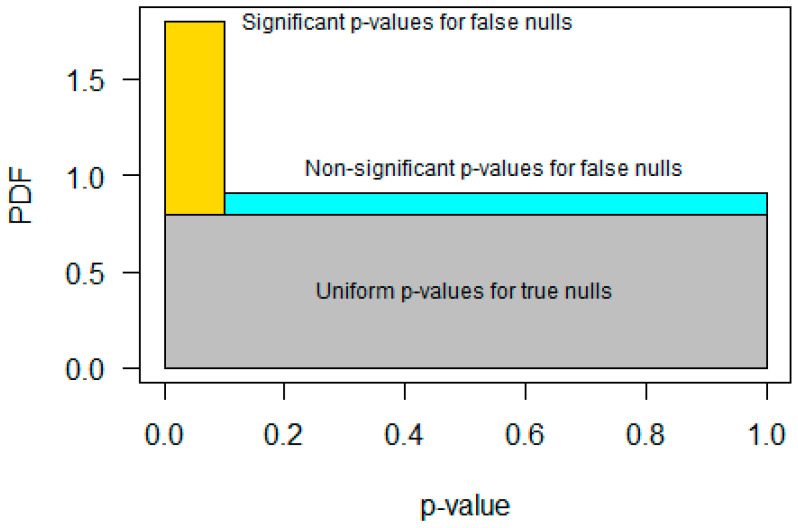
A three-rectangle approximation to the *p*-value histogram. The gray rectangle at the bottom represents the uniform distribution of p-values with a true null hypothesis. The gold rectangle at the top left represents the significant *p*-values with false null hypotheses that are less than α. The cyan rectangle at the top right represents the non-significant *p*-values with false null hypotheses that are greater than or equal α.

We now derive other estimators of ${\tilde{\pi }}_{0}$ by considering a three-rectangle histogram approximation of the distribution of *p*-values obtained by performing many hypothesis tests (Figure 1). A proportion $\pi_{0}$ of tests have a true null hypothesis. There is an average power 1−β to declare the other $1-\pi_{0}$ tests significant at the threshold α. The $\pi_{0}$ tests with a true null have uniformly distributed *p*-values by the probability integral transform. For the $1-\pi_{0}$ tests with a false null, a proportion 1−β yield *p*-values less than α and a proportion β yield *p*-values greater than α. This distribution can be approximated by three rectangles. The first is a rectangle with a height $\pi_{0}$ and base extending from p=0 to p=1; this rectangle is the uniform distribution for the proportion $\pi_{0}$ of *p*-values with a true null hypothesis (shown in gray in Figure 1). The second is a rectangle with a base extending from p=0 to p=α and a total area $\left(1-\pi_{0}\right)\left(1-\beta \right)$; this rectangle represents the 1−β subset of the proportion $1-\pi_{0}$ of *p*-values with a false null that are less than α (shown in gold in Figure 1). The height of the second rectangle is $\left(1-\pi_{0}\right)\left(1-\beta \right)/\alpha$ and its center (mean) is at p=α/2. The third rectangle has a base extending from p=α to p=1 with a total area $\left(1-\pi_{0}\right)\beta$; this rectangle represents those tests with a false null hypothesis that fail to be significant at the α level (i.e., a Type II error or what some may call a false negative result). It has a height $\left(1-\pi_{0}\right)\beta /\left(1-\alpha \right)$ and center (mean) at $p=\left(1+\alpha \right)/2$ (shown in cyan in Figure 1).

This three-rectangle approximation yields:

(6) $${\tilde{\pi }}_{hh}=\pi_{0}+\frac{\left(1-\pi_{0}\right)\beta }{1-\alpha }$$

for FDR methods such as those of [3,4,5] that estimate $\pi_{0}$ by the height of a *p*-value histogram (histogram height; HH) near p=1. It also yields:

(7) $${\tilde{\pi }}_{hm}=\pi_{0}+\left(1-\pi_{0}\right)\left(\alpha +\beta \right)$$

for FDR methods such as that of [6] that estimate $\pi_{0}$ by $2\overset{¯}{p}$; that is, twice the mean of all *p*-values (histogram mean; HM). Substituting ${\tilde{\pi }}_{hh}$ and ${\tilde{\pi }}_{hm}$ into the formula for $\tilde{\tau }$ yields simple second-order polynomials in α, with a solution provided by the quadratic formula, as described in Appendix A. These formulas for α account for the dependency of the computed FDR values on the power of the tests with false null hypotheses without the need for the computational burden of Pounds’ and Cheng’s iterative procedure [9].

Thus, with the new framework, we have derived a simple mathematical formula for the *p*-value thresholds $\alpha_{BH}$, $\alpha_{Jung}$, $\alpha_{hh},$ and $\alpha_{hm}$ for the Benjamini and Hochberg procedure [1], Jung’s formula [7], methods that use the *p*-value histogram height (HH) at p=1 to estimate $\pi_{0},$ as in Equation (6), and methods that use the *p*-value histogram mean (HM) to estimate $\pi_{0},$ as in Equation (7), respectively. These four formulas can yield different values of α (Table 1) and thus lead to different sample size estimates. Some settings in the table may seem unusual in comparison to the 80% power and 5% level calculations often performed for testing a single hypothesis. However, some applications may warrant some of these other settings. For example, one may wish to identify 100 genes with a 50% FDR (to have 50 “real” hits for follow-up) in a comparison of the expression of 10,000 genes between samples treated and untreated with a drug that alters the expression of 100 genes. This would correspond to $\tilde{\tau }=0.50$ (desired FDR), 1−β=0.50 (power to detect 50 out of 100 genes), and $\pi_{0}=0.99$ (9900 genes out of 10,000 unaltered by drug).

## 3. Supported Tests

The above section describes how to determine a threshold α for unadjusted *p*-values that yield the desired FDR $\tilde{\tau }$ and average power 1−β for a given proportion $\pi_{0}$ of tests with a true null hypothesis. The sample size to yield the desired average power 1−β at the *p*-value threshold α must still be determined using power calculation formulas for the particular statistical test of interest. Our R package performs these calculations for the following commonly applied statistical tests:

- Two-sample t-test
- One-sample t-test
- Rank–sum test
- Signed–rank test
- Fisher’s exact test
- t-test for correlation
- Comparison of two Poisson distributions
- Comparison of two negative binomial distributions
- Two-proportions z-test
- One-way ANOVA
- Cox proportional hazards regression

## 4. Algorithmic Details

The *FDRsampsize2* package uses the following general algorithm to determine the sample size:

1. Given the desired FDR τ, the desired average power 1−β, the proportion $\pi_{0}$ of tests with a true null, and the desired option for ${\tilde{\pi }}_{0}$ (BH, HH, HM, Jung), determine the *p*-value threshold α that achieves the desired FDR and average power.
2. Compute the average power for each of the two initial sample sizes $n_{0}<n_{1}$ (default $n_{0}=3$ and $n_{1}=6$; other values can be specified by the user).
3. If the average power for both of these initial sample sizes is greater than the desired average power, then report $n_{0}$ and its average power as the result.
4. If the average power for both of these initial sample sizes is less than desired, then define a new $n_{0}=n_{1}$ and $n_{1}=2n_{1}$ and compute the average power for these new $n_{0}$ and $n_{1}$. Repeat until the average power for $n_{1}$ is greater than the desired average power 1−β, and the average power for $n_{0}$ is less than the desired average power.
5. With $n_{0}$ and $n_{1}$ and their average powers as initial values, use bisection to determine the smallest n with average power greater than or equal to the desired average power 1−β.
6. To avoid excessive computing time, stop iterative calculations of steps 4 and 5 after average power has been computed a specified maximum number of times and report the results achieved thus far.

The algorithm reports the input parameters; the computed sample size (or number of events) n; the computed average power; the computed α; the number of iterations (number of times average power was computed in steps 4 and 5); the bisection bounds for the sample size $n_{0}$ and $n_{1}$; and the maximum number of iterations. If the desired average power was not achieved, the user may continue the calculations by starting at the achieved bounds for $n_{0}$ and $n_{1}$ or repeating the calculations with a greater maximum number of iterations.

## 5. Examples

### 5.1. Example 1: Study Involving Many Sign Tests

Suppose we are planning a study that will collect 10,000 Bernoulli (yes/no, positive/negative, etc.) variables for each subject. For each of those variables, we will use the sign test to evaluate the hypothesis that the probability θ of success is $\theta_{0}$ = 0.5 for each of those variables. We are interested in computing power and sample size for the setting in which 100 variables have a success probability of 0.8, and the remaining 9900 variables have a success probability of 0.5. In this setting, the null hypothesis *H*_0_: $\theta_{0}$ = 0.5 is true for 9900/10,000 ($\pi_{0}$ = 0.99) of the variables. The package includes a function *power.signtest* that uses the formula of [10] to compute the power of the sign test. The *power.signtest* is plugged into the *average.power.signtest* function. The later function computes the average power achieved by performing all tests at a fixed *p*-value level α for a given sample size and θ. The *find.sample.size* function determines the sample size needed to achieve a certain average power while controlling the FDR at a specific level.

To determine the sample size needed to achieve the desired average power that controls the FDR at τ = 0.1, we can use the *n.fdr.signtest* function directly to obtain the sample size n, computed average power, fixed *p*-value threshold, number of iterations in the function output, and the bounds of the sample size and input parameters.
theta = rep(c(0.8,0.5), c(100,9900))     # 9900 null; 100 non-nullres = n.fdr.signtest(fdr = 0.1, pwr = 0.8, p = theta, pi0.hat = “BH”)res## $n## [1] 45## ## $computed.avepow## [1] 0.8095842## ## $desired.avepow## [1] 0.8## ## $desired.fdr## [1] 0.1## ## $input.pi0## [1] 0.99## ## $alpha## [1] 0.0008879023## ## $n0## [1] 44## ## $n1## [1] 45## ## $n.its## [1] 8## ## $max.its## [1] 50

Thus, a sample size of 45 is necessary to have an average power 81%, while using the Benjamini and Hochberg procedure to control the FDR at 10% [1]. We also could obtain the sample size step-by-step; this will help users plug in the average power function of other statistical tests that are not available in *FDRsamplesize2*. As mentioned above, we first derive the *p*-value threshold α.
adj.p = alpha.power.fdr(fdr = 0.1, pwr = 0.8, pi0 = 0.99, method = “BH”)adj.p## [1] 0.0008879023

Then, we use the *find.sample.size* function to find the sample size and obtain the same output as from the *n.fdr.signtest* function above.
find.sample.size(alpha = adj.p, pwr = 0.8, avepow.func = average.power.signtest, p = theta)## $n## [1] 45## ## $computed.avepow## [1] 0.8095842## ## $desired.avepow## [1] 0.8## ## $alpha## [1] 0.0008879023## ## $n.its## [1] 8## ## $max.its## [1] 50## ## $n0## [1] 44## ## $n1## [1] 45

To compute power for a method that uses histogram height to estimate $\pi_{0}$, we specify the option *pi0.hat =* “*HH*” in *n.fdr.signtest*.
n.fdr.signtest(fdr = 0.1, pwr = 0.8, p = theta, pi0.hat = “HH”)## $n## [1] 45## ## $computed.avepow## [1] 0.810428## ## $desired.avepow## [1] 0.8## ## $desired.fdr## [1] 0.1## ## $input.pi0## [1] 0.99## ## $alpha## [1] 0.0008958549## ## $n0## [1] 44## ## $n1## [1] 45## ## $n.its## [1] 8## ## $max.its## [1] 50

These methods obtained the same sample size because $\pi_{HH}\approx 1$ in this setting with $\pi_{hh}\geq \pi_{0}=0.99$. Let us try it again with $\pi_{0}=0.95$:
theta = rep(c(0.8,0.5), c(500,9500))     # 9500 null; 500 non-nulln.fdr.signtest(fdr = 0.1, pwr = 0.8, p = theta, pi0.hat = “HH”)## $n## [1] 35## ## $computed.avepow## [1] 0.815116## ## $desired.avepow## [1] 0.8## ## $desired.fdr## [1] 0.1## ## $input.pi0## [1] 0.95## ## $alpha## [1] 0.004624029## ## $n0## [1] 34## ## $n1## [1] 35## ## $n.its## [1] 8## ## $max.its## [1] 50

In this case, we obtain the same sample size with the “BH” and “HH” options. That is not always the case. By studying Equations (6) and (7), we can see that the methods may obtain different results with smaller $\pi_{0}$, greater power, and reduced tolerance for Type I errors. For example, setting *θ* = 0.55 for tests with a false null, *θ* = 0.50 for tests with a true null, $\pi_{0}=0.90$, 1 − *β* = 0.99, and *τ* = 0.01, we obtain *n* = 3143 with the “BH” option and *n* = 3109 with the “HH” option for this problem.

### 5.2. Example 2: Design of a Clinical Trial to Find Prognostic Genes

The *FDRsamplesize2* package can also determine the sample size necessary to identify genes with expression values that are associated with progression-free survival in the context of a pediatric brain tumor clinical trial. A single-predictor Cox regression model will be used to test the association of each gene’s expression with progression-free survival (PFS). The package defines the *power.cox* function that computes the power formula of Hsieh and Lavori for Cox regression modeling [11]. This function computes power as a function of the number of events (disease progression or death). We are interested in determining the number of events necessary to identify 80% of the genes truly associated with PFS while controlling the FDR at 10% in a setting in which 1% of the genes have a hazard ratio of 2 and a variance of 1. The R code for this calculation is shown below:
log.HR = log(rep(c(1,2),c(9900,100)))   # log hazard ratio for each genev = rep(1,10000)           # variance of each generes = n.fdr.coxph(fdr = 0.1, pwr = 0.8,     logHR = log.HR, v = v, pi0.hat = “BH”)res## $n## [1] 37## ## $computed.avepow## [1] 0.8139159## ## $desired.avepow## [1] 0.8## ## $desired.fdr## [1] 0.1## ## $input.pi0## [1] 0.99## ## $alpha## [1] 0.0008879023## ## $n0## [1] 36## ## $n1## [1] 37## ## $n.its## [1] 7## ## $max.its## [1] 50

This calculation shows that 37 events provided an average power of 81.4% when using the Benjamini and Hochberg procedure to control the FDR at 10% [1]. The calculations project that with this number of events, the analysis will choose a *p*-value threshold of 0.00089 and achieve the desired average power of 80%.

### 5.3. Example 3: Differential Expression Analysis of RNA-Seq Data

Hart et al. derived a formula to compute the power of using a negative binomial model for differential expression analysis of RNA-seq data across two groups when all tests are performed at a common *p*-value threshold α [12]. They used that formula to compute the power for performing all tests at the α = 0.01 level for unadjusted *p*-values. They did not incorporate that formula into the procedure of Jung [7] or Pounds and Cheng [9].

The formula of Hart et al. expresses power as a function of sample size, the log fold change of expression, the mean read depth per gene, and the coefficient of variation per gene [12]. The *power.hart* function computes this power formula. Below, we use this function for average power calculation in the *n.fdr.negbin* to compute the sample size needed to achieve the desired average power while controlling the FDR at 10% in the setting that 99% of genes have no effect (log fold-change of 0), 1% of genes have a 2.7-fold change (log-fold change = log(2.7) = 0.99). All genes have an average read depth of 5 and a coefficient of variation equal to 0.60.
theta = log(rep(c(1,2.7),c(9900,100)))   # log fold change for each genemu = rep(5,10000)         # read depth sig = rep(0.6,10000)          # coefficient of variationres = n.fdr.negbin(fdr = 0.1, pwr = 0.8, log.fc = theta,      mu = mu, sig = sig, pi0.hat = “BH”)res## $n## [1] 20## ## $computed.avepow## [1] 0.8087842## ## $desired.avepow## [1] 0.8## ## $desired.fdr## [1] 0.1## ## $input.pi0## [1] 0.99## ## $alpha## [1] 0.0008879023## ## $n0## [1] 19## ## $n1## [1] 20## ## $n.its## [1] 6## ## $max.its## [1] 50

In this setting, with a sample size of 20 subjects per group, using the Benjamini and Hochberg procedure chooses α = 0.00089 and provides an average power of 80.9% [1].

### 5.4. Example 4: Computing Power of t-Tests for a Given Sample Size and Effect Sizes

The *FDRsamplesize2* package includes a function *fdr.avepow* that computes the FDR and average power of a set of tests as a function of α for a given sample size and effect sizes. For example, this function can compute the FDR and average power of a set of two-sample t-tests as a function of the *p*-value threshold α with n=10 per group in a setting with $\pi_{0}=0.95$ and effect size δ=2 for the remaining tests.
delta = rep(c(0,2),c(95,5)) # 95% null; 5% with delta = 2 res=fdr.avepow(10,         # per-group sample size        average.power.t.test, # average power function        null.hypo = “delta == 0”, # null hypothesis        delta = delta)     # effect size vector head(res$res.tbl) ##        alpha   fdr     pwr ## [1,] 0.001 0.02800492 0.6951602 ## [2,] 0.002 0.04869462 0.7834460 ## [3,] 0.003 0.06773070 0.8288612 ## [4,] 0.004 0.08567764 0.8577325 ## [5,] 0.005 0.10276694 0.8780755 ## [6,] 0.006 0.11912689 0.8933293

In this setting, a sample size of n=10 per group, using a fixed *p*-value threshold of α=0.005 yields an FDR of 10.3% and an average power of 87.8%.

## 6. Simulations

*FDRsamplesize2* uses published power calculation formulas for specific tests; thus, its performance is dependent on the accuracy of those power calculation formulas at the *p*-value threshold α determined by solving the equation relating the average power, the FDR, and the *p*-value threshold α. We performed a series of simulations to evaluate the accuracy of the power and sample size calculations. The simulation script is provided as a Supplementary File (*FDRsamplesize2-simulation-script.R*). The results are provided in Supplementary Table S1 (*FDRsamplesize2-simulation-results.xlsx*). Generally, the average power, FDR, and *p*-value threshold averaged over simulation replications are very close to those computed from the formulas. The calculations yielded sample sizes providing the desired FDR and average power for the two-sample *t*-test, Wilcoxon rank–sum test, comparison of two Poisson variables, *t*-test for non-zero correlations, signed–rank test, Fisher’s exact test, and one-way ANOVA.

For the few tests for which *FDRsamplesize2* yielded a sample size with inadequate FDR or average power, the power calculations were based on asymptotic normal approximations. This was the case for the two proportions z-test, the sign test, Cox regression, and the comparison of two negative binomial variables. The asymptotic normal approximations for these tests may not be sufficiently accurate for the very small *p*-value thresholds needed to maintain the desired FDR control. These results show that the accuracy of Jung’s equation relating the *p*-value threshold, average power, and FDR depends on the accuracy of the formula used to compute average power. Users should be aware of whether the underlying power calculation formulas are exact or approximate when using *FDRsamplesize2*. We designed the *FDRsamplesize2* package so that users can easily incorporate improved power calculation formulas for specific tests as they become available. It may be advisable to double-check some sample size calculations with simulation. Still, *FDRsamplesize2* provides a good starting point for using simulations to compute power and sample size.

## 7. Discussion

The FDR is a widely used metric of statistical significance in multiple-testing applications. Power and sample size calculations are an important consideration in planning studies. Formulas that relate the *p*-value threshold, FDR, average power, and the proportion $\pi_{0}$ of tests with a true null hypothesis are useful for performing these calculations. In some applications, it is important to consider how average power impacts the estimate ${\hat{\pi }}_{0}$ of $\pi_{0}$ that is used to compute an FDR estimate $\hat{\tau }$ in practice. We proposed a simple three-rectangle approximation to the *p*-value histogram to derive two new power calcualtion formulas that incorporate this consideration. We also developed the *FDRsamplesize2* R package, which performs sample size calculations using these formulas and Jung’s formula [7]. Additionally, it computes power for using the Benjamini and Hochberg procedure, which does not compute an estimate ${\hat{\pi }}_{0}$ of $\pi_{0}$ but instead effectively uses $\pi_{0}=1$ [1]. We incorporated power calculation formulas for several statistical tests not covered by other published FDR power calculation software. The *FDRsamplesize2* package also conveniently includes some individual methods scattered across different software resources, such as the method of Hart et al. to compare many negative binomial variables (read-count) across two groups [12]. Also, *FDRsamplesize2* provides a framework to compute the *p*-value threshold α needed for the desired average power and FDR, and then users can use traditional single-test power calculation methods to determine the sample size needed to achieve the desired average power at that α level. This provides additional flexibility and scope for users. Thus, *FDRsamplesize2* should be a valuable resource to researchers as they develop grant proposals and plan their experiments.

In our simulations, *FDRsamplesize2* provided very accurate sample size calculations for all tests that used exact power calculation formulas. In a few cases where asymptotic normal approximations were used to compute power, *FDRsamplesize2* yielded sample sizes that did not satisfy pre-specified requirements for the FDR or average power. The inadequate power was observed for all four methods of using $\pi_{0}$ or ${\hat{\pi }}_{0}$: the Benjamini–Hochberg approach, Jung’s equation, and the two histogram-based approaches proposed here. Thus, we encourage users to use simulation to double-check calculations based on asymptotic normal approximations for power formulas. Still, even in these cases, *FDRsamplesize2* calculations will save users time by providing useful guidance for a reasonable starting point for computationally intensive simulation-based power calculations. The Supplementary Simulation Script (*FDRsamplesize2-simulation-script.R*) also provides a useful template for performing those simulations.

There are other valuable resources to compute power and sample size for the FDR as well. The *ssize.fdr* package implements the method of Liu and Hwang for two-sample *t*-tests and some ANOVA tests [13,14]. The PROPER method was designed specifically for planning differential expression analyses of RNA-seq data [15]. Hu et al. developed a method using the expectation–maximization algorithm to compute power and sample size [16]. Shao and Tseng developed a method to carefully account for correlation among tests in computing power and sample size [17]. Pawitan et al. developed a method for performing many two-sample *t*-tests [18]. The *scPower* framework computes power for multi-sample single-cell transcriptomic experiments [19]. Ching et al. provided a simulation-based framework to compute power for RNA-seq differential expression analysis [20]. Jung et al. developed a method to compute sample size for a one-way blocked design [21]. Lee and Whitmore provided a framework to compute power for elaborate linear models [22]. Li et al. developed a bulk method for performing exact tests on RNA-seq data. All these methods are approximations that work well when a very large number (hundreds or more) of hypothesis tests are performed [23]. Glueck et al. developed a method to compute the *exact* power of the Benjamini and Hochberg procedure [1] for applications involving a relatively small number of hypothesis tests (such as evaluating multiple co-primary endpoints in a clinical trial) [24]. Numerous tools are now available for many specific study designs and scientific objectives in the literature. Certainly, there are still other applications for which power calculation methods need to be developed. These provide opportunities for those interested in developing power calculation methods for planning genomic studies.

## Figures and Tables

**Table 1:** Example calculations of α.

$\pi_{0}$	τ	1−β	$\alpha_{BH}$	$\alpha_{hh}$	$\alpha_{hm}$	$\alpha_{Jung}$
0.90	0.05	0.5	0.002618	0.002762	0.002762	0.002924
0.95	0.05	0.5	0.001312	0.001348	0.001348	0.001385
0.99	0.05	0.5	0.000263	0.000264	0.000264	0.000266
0.90	0.10	0.5	0.005495	0.005812	0.005810	0.006173
0.95	0.10	0.5	0.002762	0.002841	0.002840	0.002924
0.99	0.10	0.5	0.000555	0.000558	0.000558	0.000561
0.90	0.50	0.5	0.045455	0.049740	0.049510	0.055556
0.95	0.50	0.5	0.023810	0.024968	0.024938	0.026316
0.99	0.50	0.5	0.004950	0.005000	0.005000	0.005051
0.90	0.05	0.8	0.004188	0.004571	0.004569	0.004678
0.95	0.05	0.8	0.002100	0.002192	0.002192	0.002216
0.99	0.05	0.8	0.000421	0.000424	0.000424	0.000425
0.90	0.10	0.8	0.008791	0.009636	0.009627	0.009877
0.95	0.10	0.8	0.004420	0.004624	0.004623	0.004678
0.99	0.10	0.8	0.000888	0.000896	0.000896	0.000898
0.90	0.50	0.8	0.072727	0.084772	0.083619	0.088889
0.95	0.50	0.8	0.038095	0.041201	0.041063	0.042105
0.99	0.50	0.8	0.007921	0.008048	0.008047	0.008081

## Data Availability

Data are contained within the article and Supplementary Materials.

## Supplementary Materials

The following supporting information can be downloaded at: https://www.mdpi.com/article/10.3390/genes15030344/s1, Table S1: Results of Simulation Studies for desired power = 0.5 and desired FDR = 0.05.

## Appendix A

This appendix provides details on solving the $\tilde{\tau }$ equation for α with ${\tilde{\pi }}_{0h}$ (histogram height) and ${\tilde{\pi }}_{0m}$ (histogram mean).

1. Histogram Height

With ${\tilde{\pi }}_{0h}=\pi_{0}+\frac{\left(1-\pi_{0}\right)\beta }{1-\alpha }$ (dropping the tilde notation for simplicity), we have:

$$\tau =\frac{\left(\pi_{0}+\left(1-\pi_{0}\right)\beta /\left(1-\alpha \right)\right)\alpha }{\pi_{0}\alpha +\left(1-\pi_{0}\right)\left(1-\beta \right)}$$

which can be equivalently expressed as:

$$\tau \pi_{0}\alpha +\tau \left(1-\pi_{0}\right)\left(1-\beta \right)=\left(\pi_{0}+\frac{\left(1-\pi_{0}\right)\beta }{1-\alpha }\right)\alpha$$

and then as:

$$\tau \pi_{0}\alpha \left(1-\alpha \right)+\tau \left(1-\pi_{0}\right)\left(1-\beta \right)\left(1-\alpha \right)=\pi_{0}\alpha \left(1-\alpha \right)+\left(1-\pi_{0}\right)\beta \alpha$$

and finally, as:

$$-\left(1-\tau \right)\pi_{0}\alpha^{2}+\left(\tau \left(1-\pi_{0}\right)\left(1-\beta \right)+\left(1-\tau \right)\pi_{0}+\left(1-\pi_{0}\right)\beta \right)\alpha +\tau \left(1-\beta \right)\left(1-\pi_{0}\right)=0$$

which is solved as:

$$\alpha =\frac{-b+\sqrt{b^{2}-4ac}}{2a}$$

with:

$$a=-\left(1-\tau \right)\pi_{0},\;b=\tau \left(1-\pi_{0}\right)\left(1-\beta \right)+\left(1-\tau \right)\pi_{0}+\left(1-\pi_{0}\right)\beta ,\;\mathrm{and}\;c=\tau \left(1-\beta \right)\left(1-\pi_{0}\right).$$

2. Histogram Mean

Similarly, with ${\tilde{\pi }}_{0m}=\pi_{0}+\left(1-\pi_{0}\right)\left(\alpha +\beta \right)$, we obtain:

$$\tau =\frac{\left(\pi_{0}+\left(1-\pi_{0}\right)\left(\alpha +\beta \right)\right)\alpha }{\pi_{0}\alpha +\left(1-\pi_{0}\right)\left(1-\beta \right)}$$

which can be expressed as:

$$\tau \pi_{0}\alpha +\tau \left(1-\pi_{0}\right)\left(1-\beta \right)=\pi_{0}\alpha +\left(1-\pi_{0}\right)\left(\alpha +\beta \right)\alpha$$

and then as:

$$\left(1-\pi_{0}\right)\alpha^{2}+\left(\left(1-\tau \right)\pi_{0}+\left(1-\pi_{0}\right)\beta \right)\alpha -\tau \left(1-\pi_{0}\right)\left(1-\beta \right)=0$$

which is solved as:

$$\alpha =\frac{-b+\sqrt{b^{2}-4ac}}{2a}$$

with:

$$a=\left(1-\pi_{0}\right),\;b=\left(1-\tau \right)\pi_{0}+\left(1-\pi_{0}\right)\beta \;\mathrm{and}\;c=-\tau \left(1-\pi_{0}\right)\left(1-\beta \right).$$
